# Validation of the Cognitive Reserve Index Questionnaire (CRIq) in Arabic

**DOI:** 10.3390/bs13121006

**Published:** 2023-12-10

**Authors:** Natali Farran, Hala Darwish

**Affiliations:** 1 Department of Psychology, Institute of Psychiatry, Psychology and Neuroscience, King’s College London, London SE5 8AB, UK; 2 Hariri School of Nursing, American University of Beirut, Beirut 11-0236, Lebanon; 3 Department of Systems, Populations and Leadership, School of Nursing and Department of Neurology, School of Medicine, University of Michigan, Ann Arbor, MI 48109, USA

**Keywords:** cognitive reserve, Cognitive Reserve Index Questionnaire (CRIq), Arabic, Lebanon, psychometric validation

## Abstract

Cognitive reserve is the adaptability of cognitive processes in the face of brain aging and pathology. This study aimed to validate the Arabic version of the Cognitive Reserve Index Questionnaire (CRIq) in a healthy Lebanese sample. CRIq assesses cognitive reserve through three domains: education, working activity, and leisure time. Statistical measures, including descriptive and regression analysis along with structural equation modeling, were utilized to investigate the convergent and discriminant validity of the CRIq, incorporating fluid intelligence (Gf) and measures of cognitive function, long-term memory encoding and retrieval (Glr), and processing speed (Gs). Results from 174 participants revealed that the activities assessed by the CRIq-Arabic were comparable to the original CRIq study, with slight cultural differences. The internal consistency of the CRIq-Arabic was good (Cronbach’s α = 0.88), indicating reliability. Convergent validity was confirmed, with moderate to high loadings on the cognitive reserve latent construct. Discriminant validity was supported as correlations between cognitive reserve variables and non-target constructs (Gf, Glr, and Gs) were less than 1. The findings provide an initial psychometric validation of the CRIq-Arabic. Further research of clinical samples is needed to enhance its utility in neuropsychological practice.

## 1. Introduction

The disjunction between the degree of brain pathology and its clinical manifestations gave rise to the term “cognitive reserve or brain reserve” [1]. These constructs often mediate between clinical manifestations and the extent of brain pathology [2]. Cognitive reserve refers to “the adaptability (i.e., efficiency, capacity, flexibility) of cognitive processes that help to explain differential susceptibility of cognitive abilities or day-to-day function to brain aging, pathology, or insult” [3]. It includes determinants such as educational and occupational attainments [4,5] and is suspected to comprise a functional network actively involved in diverse cognitive processes [6]. Cognitive reserve has been investigated in many clinical populations with neurocognitive disorders, including Alzheimer’s disease [7], Multiple Sclerosis [8], Schizophrenia [9], movement disorders such as Parkinson [10], Huntington’s disease [11], and HIV [12]. The construct is vital for diagnostic purposes [2] and is a promising avenue for preventing and intervening in cognitive decline [13,14,15]. Some also promote it as a population-level intervention [16]. Currently, a reliable and valid tool for estimating cognitive reserve in the Arabic language is absent, as opposed to other languages such as Italian [17], Spanish [18], Greek [19], and English [20].

There are five approaches to estimating cognitive reserve: (I) measurement of individual characteristics, (II) consideration of cumulative life experiences, (III) estimation of intellectual functioning, (IV) statistical modeling and calculations, and (V) derivation of brain network patterns via imaging [2]. Each approach has its advantages and disadvantages—which will most likely change with time. For instance, implementing statistical methods (decomposing the variance of a specific cognitive skill) provides an operational measure of reserve that is quantitative, continuous, and specific to the individual. However, it remains not feasible for the clinician to apply such scores individually [21]. Approach II, on the other hand, synthesizes numerous experiences relevant to the cognitive reserve construct and is currently feasible [1,22]. The instruments which adopt such an approach are the following: the Cognitive Reserve Index Questionnaire (CRIq) [17], the Cognitive Reserve Questionnaire (CRQ) [23], the Cognitive Reserve Scale (CRS) [18], the Lifetime of Experience Questionnaire (LEQ) [20], the Retrospective Indigenous Childhood Enrichment scale (RICE) [24], the Premorbid Cognitive Abilities Scale (PCAS) [25], and the Cognitive Reserve Assessment Scale in Health (CRASH) [26]. For a comprehensive review of these scales (except for CRASH), the reader is referred to [22]. Although the authors did not draw a recommendation for one specific tool, they indicated that the CRIq is most extensively evaluated [22]. Compared to other tools, the CRIq balances out between administration time, cognitive reserve dimensions covered, interview span, and psychometric properties [17,22].

We previously examined cognitive reserve in Lebanon using approach I (i.e., measurement of individual characteristics) [27]. In a sample of 508 community-based older adults, we showed that high education, complex occupation attainment, and leisure activity significantly predicted better global cognitive function [27]. The current study aims to analyze the psychometric properties of the Arabic version of the CRIq. Since cognitive reserve focuses on the idea that there are individual differences in the adaptability of functional brain processes that allow some people to cope better than others with age- and disease-related brain change [1,2,3], we adopt an individual differences approach in validating the tool [28,29,30]. We hypothesized the following: (I) CRIq-Arabic activities would slightly differ from the original study (due to a variety of factors such as cultural and demographic variances), (II) the CRIq-Arabic would show good internal consistency properties, (III) the cognitive reserve domains (education, working activity, and leisure time) as measured by the CRIq-Arabic would converge with cognitive reserve as a latent construct, and (IV) the cognitive reserve constructs would stand out from other functional/cognitive processes and diverge from measures of cognitive functioning (such as fluid intelligence).

## 2. Materials and Methods

The cross-sectional observational study is part of a larger project to validate an Arabic version of the Brief International Cognitive Assessment for Multiple Sclerosis (BICAMS) [31]. All participants provided written informed consent.

### 2.1. Sample

Individuals from the general community in Lebanon were approached and recruited for the larger study (non-clinical sample from various areas in Lebanon and those meeting our inclusion and exclusion criteria) [31]. We purposefully sampled the study participants in accordance with relevant socio-demographic characteristics of individuals in Lebanon (age range distribution, starting at the age of 16 years, and educational attainment). According to data from the Central Intelligence Agency (CIA) in 2020, the literacy rate is 99.24% for individuals aged 15 to 24 years old and 60.15% for those aged 65 and above [32]. The population breakdown in Lebanon is as follows: 16% are between 15 and 24 years old, 45.27% are aged 25 to 54, and 8.3% and 7% are 55–64 and 65 years or older, respectively, with an expected 11–12 years of schooling. Additionally, in 2017, reports showed that 93% of the population completed primary education, 63–70% finished secondary education, and 45–49% attained tertiary education [33].

Individuals were recruited for the larger project using different methods including flyers posted at the hospital, clinics, and various social media platforms. However, we found that relying on word-of-mouth and snowball techniques was more efficient, and the majority of the final sample was recruited from the community through these methods. Two screening phases were run to ensure that the sample was healthy. For the parent study, during the first phase, participants were excluded if they were younger than 16 years and had a history of neurological disorders, traumatic brain injury, or psychiatric disorders (including alcohol and/or drug dependence). Individuals were also excluded if taking medications affecting cognitive function, such as antipsychotics or antidepressants. During the second screening phase, participants were excluded if they had symptoms of depression or cognitive difficulties. The former was assessed using the Hopkins Symptoms Checklist-25 (HSCL-25; 3.3 cut-off score) [34,35,36] and the latter using the Montreal Cognitive Assessment (MoCA; cut-off scores: 26 for individuals < 60 years of age and 24 for those ≥ 60 years of age) [37,38,39]. In this study, we limited our sample to data from participants aged 18–80 years. The lower age group was based on the original CRI-q study [17].

### 2.2. Procedure and Data Collection

We followed the World Health Organization guidelines on translation and adaptation of instruments [40]; translated the CRIq to Modern Standard Arabic (MSA), which is used in formal and written standards across the Arab world; and had the tool back-translated by an experienced English instructor who teaches at a university level. The principal investigator reviewed the tool and adapted two questions based on cultural Lebanese factors. The first is that working as a nurse was considered professional employment. The second is that the fourth question in CRIq-Arabic leisure time included additional hobbies such as Backgammon and playing cards (common in the Lebanese culture).

The CRIq is a semi-structured interview that includes 20 items and demographic information. It takes approximately 15 min to be administered. The instrument examines the frequency and duration of the three sets of activities—education (years of formal and informal education), working activity (years and level of professional occupation), and leisure time (years of frequent attainment of various activities such as reading books). As such, the CRIq includes an index score and a score for each of its three domains. These scores are adjusted for age [17].

To examine fluid intelligence, we used the Test of Nonverbal Intelligence (TONI-III) [41]. The TONI-III is a language-free intelligence test that includes 45 items to be solved (for each form). Ceiling/discontinuation rules apply to the test, and raw scores are normed to an American sample of 3451 individuals [41].

The Arabic BICAMS includes three tests: the Symbol Digit Modalities Test (SDMT), the Brief Visuospatial Memory Test—Revised Edition (BVMT-R), and the Verbal Memory Arabic Test [31]

The SDMT examines processing speed. The oral version of the test was administered. Using a test form containing nine symbol-digit pairings (key) and a pseudo-randomized sequence of symbols (stimuli), the examinee must respond by voicing the digit associated with each symbol as quickly as possible. A sequence of 10 symbols is first used for practice. Then, the participant is given 90 s to complete as many items as possible present in the form after the practice items. The score reflects the number of correct responses [42].

For the BVMT-R, participants are exposed for 10 s to a matrix of six simple abstract designs followed by an unaided recall. The examinee is asked to reproduce the designs using paper and pencil as accurately as possible and to place the figures in their correct positions. In total, three such trials occurred for each participant. Each figure can receive a 0, 1, or 2 score based on accuracy and location scoring criteria [43]. The score of interest was the total of trials 1 to 3.

We recently developed and validated a Verbal Memory Arabic Test (VMAT), which substituted the California Verbal Learning Test-2nd Edition (CVLT-II) in our study (the former is part of the original BICAMS protocol). The VMAT was developed indigenously in Arabic using quantitative and qualitative methods. The instrument measures verbal learning, short-term memory, long-term memory, and recognition. Similar to other standardized verbal learning/memory tests, and in line with Benedict et al’s. [43] recommendations, the examinee is presented with 15 words (List A) to be recalled freely across five trials and is then presented with another 15 words (List B) which serve as an interference trial. Following the recall of List B, the participant is required to recall List A with and without semantic cues. Following a 25 min delay, the test-taker must recall List A with and without cues and then recognize the words from List A from an array of 45 words that include List A, List B, and additional distractors. Several scores can be derived from the VMAT [44]. The VMAT variables used in this study were the total number of words recalled on trials 1 to 5, short delay-free recall, and long delay-free recall.

All tests were administered in a standardized manner, in a quiet room, using the Lebanese Arabic dialect, which is the spoken Arabic form used in everyday communication (the CRI-q was in Modern Standard Arabic). After the screening phases, the CRIq was administered, followed by the Test of Nonverbal Intelligence, 3rd Edition (TONI-III) and then the BICAMS.

### 2.3. Statistical Analysis

Descriptives of demographic data, scores on cognitive measures, and CRIq-Arabic questions were derived. Specifically, for the CRIq-Arabic education domain, information on the average number of years for the two education items is presented. For the CRIq-Arabic working activity domain, percentages of types of working activity according to cognitive resources involved are listed. For the CRIq-Arabic leisure time domain, percentages of types of activities carried out during leisure time are indicated. Descriptive data for the three CRIq-Arabic domains are also derived according to each age group (young, middle-aged, and older adults).

CRIq-Arabic standardized scores were derived using information from regression models. Each CRIq-Arabic domain was subjected to this analysis with age as an independent variable. Assumptions such as homoscedasticity and independence of observations were checked as appropriate. None of the models violated the assumptions.

For reliability, internal consistency was examined using Cronbach’s α and split-half correlations between odd and even questions of the CRIq-Arabic.

Structural equation modeling (SEM) was used to examine convergent and discriminant validity. We built four models following the Salthouse et al. (2003) approach and based on the previous literature [29,30], as well as data availability. The larger BICAMS project did not include explicit measures of executive functions such as mental flexibility. The cognitive domains present were based on the Cattell–Horn–Carroll Model of cognition [45,46]. Fluid reasoning (Gf) was reflected through TONI-III. Long-term memory encoding and retrieval (Glr) was reflected through VMAT’s scores on total trials 1 to 5, short delay-free and long delay-free recalls, as well as the total score on BVMT-R for trials 1 to 3. Processing speed (Gs) was reflected through the SDMT score.

**Figure 1:**
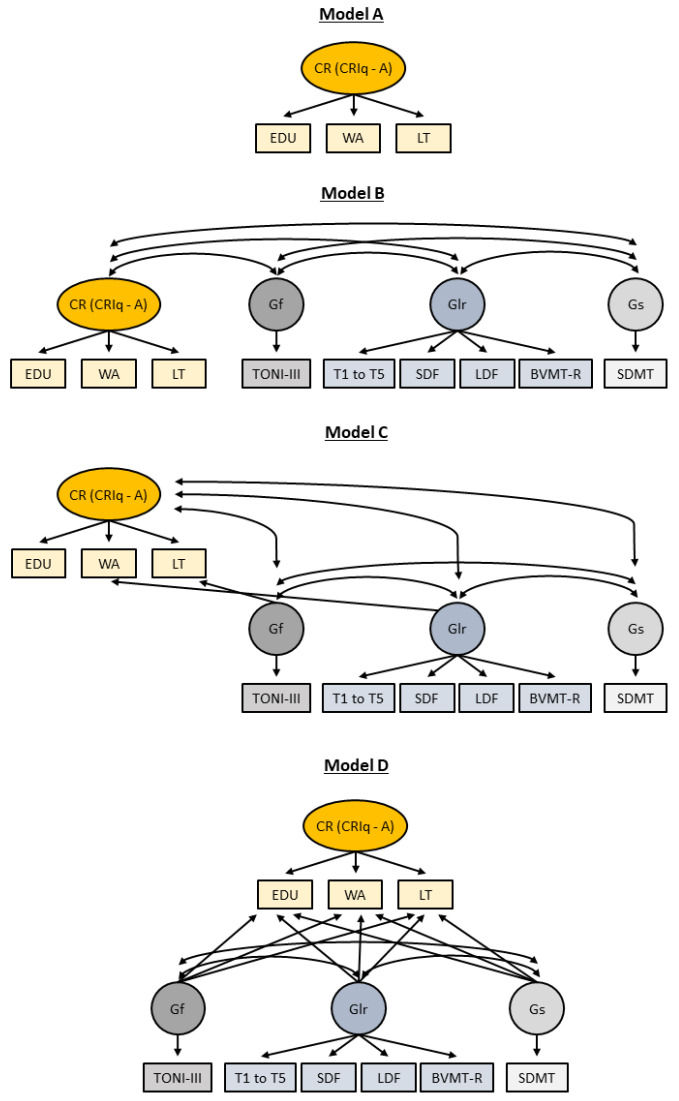
Models for construct validity of CRIq-Arabic. Gf: fluid intelligence, Glr: long-term memory encoding and retrieval, Gs: processing speed, CRIq-A: Cognitive Reserve Index Questionnaire—Arabic., EDU: education, WA: working activity, LT: leisure time, TONI-III: Test of Nonverbal Intelligence, 3rd Edition, T1 to T5: Verbal Memory Arabic Test total trials 1 to 5, SDF: Verbal Memory Arabic Test short delay-free, LDF: Verbal Memory Arabic Test long delay-free, BVMT-R: Brief Visuospatial Memory Test—Revised Edition, SDMT: Symbol Digit Modalities Test.

Figure 1 depicts the four models.

The modeling approach was a graded one and progressed towards more demanding tests of construct validity [28,30]. It aimed to examine whether the cognitive reserve construct measured using the tool CRIq-Arabic is coherent. More specifically, the approach looked at whether the variables that were used to represent cognitive reserve, which the CRIq-Arabic measured, are related to one another and that they are different from other known cognitive domains (such as memory). Four models were built: A, B, C, and D. The elements looked at in the models were convergent and had discriminant validity. The former relates to understanding if the variables—education, working activity, and leisure time, which represent cognitive reserve, the latent construct—have significant variances in common (using the factor loadings in the model). Convergent validity is established through moderate to strong loadings on the hypothesized construct. The latter relates to evaluating how cognitive reserve, as measured by the CRIq-Arabic, is related to cognitive domains (such as Gf). It is established through weak relations, correlations, and loadings between these domains [28].

Model A includes only the CRIq-Arabic target variables (education, working activity, and leisure time) and the hypothesized construct (CRIq-Arabic). This model is a first step and looks at whether there is a significant variance between the variables that make up cognitive reserve (e.g., working activity).

Model B then adds to the first model by allowing the hypothesized construct to be related to the different cognitive areas included in the study (Gf, Glr, and Gs).

Model C also allows the target variables that make up the hypothesized cognitive reserve to be related to other cognitive constructs if they result in an improved fit compared to Model B.

Lastly, Model D examines variance common to the hypothesized construct, i.e., cognitive reserve, when the relations of individual variables with other cognitive domains are considered (such as Gf).

Full-information maximum likelihood estimation was used to deal with missing data. To achieve identifiability, parameters for CRIq-Arabic leisure time were fixed at one. Reliability was set at 0.8 for TONI-III and SDMT to correct for single indicator constructs. The values selected were based on literature that shows good psychometric properties of these tests [42,47].

The fit of the models was examined using several parameters, which were chi-square (X2), the critical ratio (X2/df), the root mean square error of approximation (RMSEA), and Bentler’s comparative fit index (CFI). For CFI, values closer to one indicated a good model fit. For other parameters, values closer to zero indicated a good model fit [48].

Analyses were performed on SPSS version 25 and AMOS version 23. Results with *p* < 0.05 were set as significant, and two-tails were used.

## 3. Results

### 3.1. Participants

Some 226 individuals between the ages of 18 and 80 years were approached. Of these, 23 were excluded during the first screening and 29 during the second screening. Within the latter, 25 individuals were screened out due to the presence of cognitive difficulties as assessed by the MoCA. The final sample included 174 participants with a mean age of 44.40 ± 18.37. Sample characteristics are found in Table 1, and scores on cognitive measures are in Table 2.

### 3.2. CRIq-Arabic Descriptions and Computations

Descriptive data about the CRIq-Arabic activities performed are depicted in Table 3.

**Figure 2:**
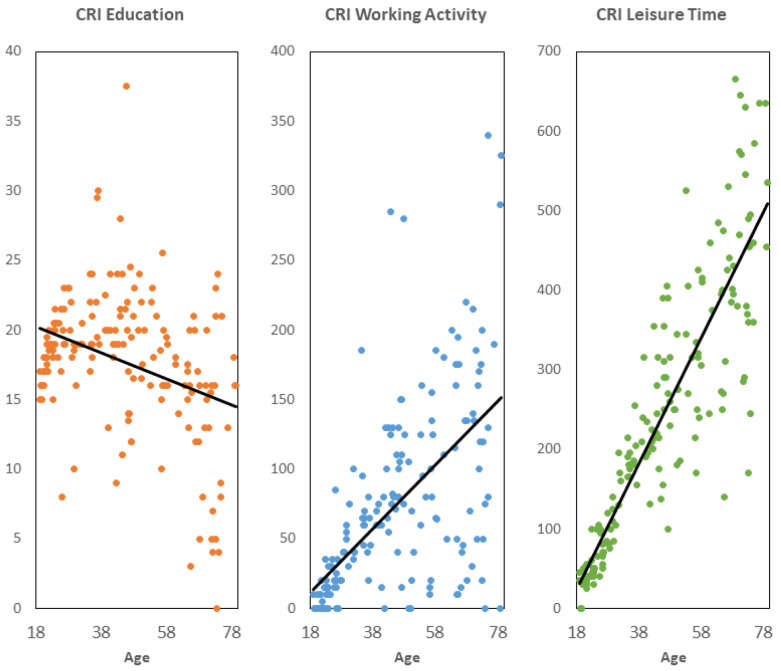
Scatter plots of raw scores of the three cognitive reserve domains by age.

The mean raw scores for the CR domains of education, working activity, and leisure time were 17.75 ± 4.99, 71.76 ± 70.15, and 232.67 ± 164.95, respectively. Figure 2 includes a scatter plot for each domain of raw scores by age.

All regression models, whereby age was a predictor and each CR domain was a dependent variable, were significant (*p* < 0.001). For the CR domain education, the y-intercept was 21.86 and the slope was—0.09. This yielded average age-corrected scores that ranged from 54.79 to 159.71, with a mean of 100 and an SD of 14.10.

For the domain of working activity, the corrected scores ranged from 67.78 to 145.89 (100 ± 12.02) after a y-intercept of—29.67 and a slope of 2.28.

For the domain of leisure time, corrected scores ranged between 73.59 and 121.50 (100 ± 7.02), following a y-intercept of—119.58 and a slope of 7.93.

The final CR index ranged from 47.39 to 149.52 (100 ± 15). An Excel file for automatic computations is available from the authors upon request.

### 3.3. Internal Consistency

Measures of internal consistency showed good evidence of reliability. Cronbach’s α of the full scale was 0.88. The correlation between the odd and even forms was 0.80 for the split-half procedure, and the Spearman–Brown coefficient was 0.89.

### 3.4. Construct Validity

Figure 1 depicts the models, and Table 4 includes the results.

A one-factor model composed of education, working activity, and leisure time as target variables, and the cognitive reserve latent construct, measured by CRIq-Arabic, was run for Model A. The model’s overall fit could not be determined since there were no degrees of freedom. Nonetheless, the three CRIq-Arabic variables had significant moderate to solid loadings on the latent construct. Therefore, initial evidence for convergent validity is present.

Model B examines the latent construct in the context of three non-target constructs: Gf, Glr, and Gs. Correlations between the target and non-target variables were substantially less than 1 (e.g., Gs = 0.37), thus showing initial evidence of discriminant validity. The model also continued to show that the variables hypothesized to represent cognitive reserve have convergent validity with moderate to high loadings. The model fit characteristics were adequate.

Two non-target constructs (i.e., Gf and Glr) can relate to two cognitive reserve variables in Model C. The model’s overall fit did not change compared to Model B. Evidence of convergent validity continued to be present with moderate to high loadings (e.g., working activity = 0.64). For discriminant validity, correlations slightly increased but were still significantly less than 1 (e.g., Gs = 0.48).

In Model D, each non-target construct is allowed to relate to each of the observed cognitive reserve variables simultaneously. Loadings became moderate, but they were low for education (0.35). From the non-target variables, only Gf loaded significantly with education; estimate = 0.27.

## 4. Discussion

This study examined the psychometric properties of an Arabic version of the CRIq. The world’s fifth most spoken language is Arabic, and 23 countries have Arabic as their official language [49]. Results of our study suggest that the performed activities are comparable to the original CRIq study and that the tool exhibits good construct validity and internal consistency. Integrating a valid cognitive reserve measure into the diagnostic formulation is critical [2]. Some clinical considerations include—but are not limited to—differences between individuals with high versus low reserve when clinical symptoms are demonstrated, cognitive help as a factor affecting the rate of decline, and the possibility of it being a factor that influences response to treatment [2]. 

Education and the percentages of types of activities carried out within the working activity domain and leisure time domain were primarily comparable to Nucci et al. [17]. There were slight differences, however, in the distribution of a few types of activities, which is partly in concordance with our first hypothesis. This highlights the importance of relying on culturally appropriate data [2,50]. Specifically, level 1 working activity (i.e., low-skilled manual work) was higher in Nucci et al. [17] as opposed to our sample, in which level 4 was the highest (i.e., professional occupation). Furthermore, the two leisure time activities that differed were ‘using new technologies’ and ‘managing one’s bank account’. While both activities decreased with age, in our sample, they remained higher than the ones performed by Nucci et al. [17]. In addition to some apparent lifestyle differences between both samples, it should be noted that individuals in our sample were screened out in case of cognitive impairment, as assessed by the MoCA. Nucci et al. indicated that their sample had no evident neurologic or psychiatric illness [17]. However, it was unclear if individuals were screened based on cognitive functioning. Subsequently, given the difference in the distribution of work activity between both samples, the sample profile could explain part of the differences. It should be noted that caution must be exercised when using the CRIq-Arabic scale with individuals with limited educational attainment (as the models showed a significant loading of educational attainment in cognitive reserve). Most individuals in this study have attained a college degree (uneven distribution of educational levels), thus limiting the generalizability of our results.

The measure of internal consistency (Cronbach’s α = 0.88) was good in our study and higher than Nucci et al. [17] Cronbach’s α = 0.62). This supports one form of CRIq-Arabic reliability and fulfills our second hypothesis. Further studies are needed to establish other types of reliability of the Arabic version (e.g., test–retest).

Theoretically, examining cognitive reserve has several measurement challenges [16,51]. Endorsing these challenges, we resorted to validating the CRIq-Arabic using the approach followed by Salthouse et al. [28] and applied by others in cognitive reserve [29,30]. Our models consistently showed that the cognitive reserve domains, education, working activity, and leisure time, as measured by the CRIq-Arabic, converge with the latent construct of cognitive reserve, fulfilling the third hypothesis. This result is not surprising given the ample evidence on the role of these variables in cognitive reserve [4] and their utility in most cognitive reserve scales [22]. For example, the LEQ examines specific (education, occupation) and non-specific mental activity (leisure time) for each lifespan (three stages) [20]. In addition to Nucci et al. [17], this result supports one aspect of construct validity of the CRIq in general. To the best of our knowledge, only Nucci et al. [17] previously examined the convergent validity of the scale [22]. The result is likewise in concordance with our previous study in Lebanon, in which education, complex occupation attainment, and leisure activity significantly predicted better global cognitive function in a sample of older adults [27].

Lastly, there was good support for the fourth hypothesis, which indicated that the cognitive reserve construct would diverge from measures of cognitive functioning (such as fluid intelligence). Ideally, as a first step to provide evidence of discriminant validity, the magnitude of the loadings on the cognitive reserve construct from the observed variables (in this study, education, working activity, and leisure activity) should be the same or larger than the correlations among the target and non-target constructs (in this study, Gf, Glr, and Gs) [28]. This was the case in Models B and C. Results with Model D, which is the most stringent compared to Models A, B, and C [28], suggest that education is related to Gf. It is difficult to compare this result with other studies since none examined Gf [29,30,52], although Satz et al. [51] implicate fluid cognitive ability in cognitive reserve. More comprehensive studies that utilize measures of fluidity (in addition to crystallized intelligence) can better inform cognitive reserve measurement. It should also be noted that while education is one of the valid proxy indicators of cognitive reserve, some suggest that literacy is a more sensitive indicator [53,54].

Our findings should be interpreted considering several limitations. The main limitation is that executive functions were not measured. Although the contribution of executive functions to unique variance in cognition is debated [45,55], this construct remains elusive in cognitive reserve [28,51,52]. The second limitation is that the scale was not validated in a clinical sample. Indeed, Cosentino and Stern [2] indicate that the concept of cognitive reserve only applies when considering variability in cognitive functioning (i.e., memory) in the face of changes in brain integrity (i.e., hippocampal volume). Future studies on CRIq-Arabic validation should be performed in clinical populations such as those with Multiple Sclerosis. Other limitations include the lack of Arabic normative data for most of the used scales and the utility of one measure for Gf, as well as Gs. Despite the sample size being comparable to other research on CRIq [22], and to a study that tested formative and reflective models in cognitive reserve [29], it remains small when compared to other studies such as the ones by Nucci et al. [17] and Siedlecki et al. [30]. Despite these limitations, our study showed strength in its utility of theoretically driven models and in applying rigorous-construct validity testing.

This study validated one of the most used cognitive reserve scales in Arabic in a healthy Lebanese sample (the scale was not examined in other Arab countries). Further validation studies in clinical samples, larger healthy samples, and more diverse individuals in terms of educational attainment are warranted for additional scale evaluation and usability in other contexts. Since the tool is in an MSA format, it can be used in various Arab countries with further examination of its psychometric properties in such samples (and not just limited to Lebanese individuals). The CRIq-Arabic can be valuable in enhancing neuropsychological clinical practice, and the results of our study are encouraging in terms of initial steps in psychometric validation.

## Figures and Tables

**Table 1:** Demographic information.

Age Group	F	%
Young adults (18–35 years)	69	39.66
Middle-aged adults (36–55 years)	50	28.74
Older adults (56–80 years)	55	31.61
**Sex**		
Male	68	39.08
Female	106	60.92
Education *		
Literate with no schooling	2	1.15
*Young adults*	*0*	*0*
*Middle-aged adults*	*0*	*0*
*Older adults*	*2*	*1.15*
Elementary to Intermediate	15	8.62
*Young adults*	*0*	*0*
*Middle-aged adults*	*2*	*1.15*
*Older adults*	*3*	*7.47%*
Some high school	7	4.02
*Young adults*	*0*	*0*
*Middle-aged adults*	*4*	*2.30*
*Older adults*	*3*	*1.72*
Completed high school	24	13.79
*Young adults*	*12*	*6.90*
*Middle-aged adults*	*4*	*2.30*
*Older adults*	*8*	*4.60*
Vocational education	12	6.90
*Young adults*	*1*	*0.57*
*Middle-aged adults*	*3*	*1.72*
*Older adults*	*8*	*4.60*
University	114	65.52
*Young adults*	*56*	*32.18*
*Middle-aged adults*	*37*	*21.26*
*Older adults*	*21*	*12.07*
Current employment status		
Employed	100	57.47
Unemployed	74	42.53
Marital status		
Married	75	43.10
In a relationship	12	6.90
Divorced, separated, or widowed	21	12.07
Single	66	37.93
General health		
Illness present (apart from the health-related exclusion criteria) **	46	26.44
Illness absent	128	73.56
Smoking		
Yes	50	28.74
No	124	71.26
Physical activity		
Yes	125	71.84
*Frequency*		
*1–2 times per week*	*38*	*21.84*
*3–4 times per week*	*32*	*18.39*
*5 or more times per week*	*47*	*27.01*
*1–5 times per month*	*8*	*4.60*
No	49	28.16

F: Frequency. * Percentages per age group are from the total sample. ** From these, 11 individuals had diabetes (6.32% of the total sample), 7 had hypertension (4.02%), and 7 had both diabetes and hypertension (4.02%); other individuals in the sample who had an illness varied in these (such as those with migraines or asthma).

**Table 2:** Scores on cognitive measures.

Gf	M	SD
Test of Nonverbal Intelligence, 3rd Edition	103.02	16.82
**Glr**		
Verbal Memory Arabic Test: Trials 1 to 5	50.90	9.19
Verbal Memory Arabic Test: Short Delay-Free	10.27	2.76
Verbal Memory Arabic Test: Long Delay-Free	10.72	2.60
Brief Visuospatial Memory Test—Revised	22.25	6.95
**Gs**		
Symbol Digit Modalities Test	55.38	13.28

M: mean. SD: standard deviation. Gf: fluid intelligence. Glr: long-term memory encoding and retrieval. Gs: processing speed.

**Table 3:** Descriptives of CRIq-Arabic activities performed.

Cognitive Reserve Domain	Total Sample	Young Adults	Middle-Aged Adults	Older Adults
Education *				
Years of education (including postgraduate/ specializations)	17.09 ± 4.83	18.36 ± 2.75	18.93 ± 4.83	13.82 ± 5.28
Years of vocational training	10.66 ± 1.31	0.26 ± 0.52	1.07 ± 1.78	0.79 ± 1.39
Working activity **				
Never employed	0.71	1.69	0.09	0.59
Level 1: low-skilled manual work	4.01	3.55	2.38	8.89
Level 2: skilled manual work	21.84	30.51	14.53	27.17
Level 3: skilled non-manual work	25.63	30.67	23.40	23.72
Level 4: professional occupation	39.77	27.31	49.20	34.80
Level 5: highly responsible or intellectual occupation	8.04	6.27	10.40	4.83
Leisure time **				
Reading newspapers and magazines	7.26	7.42	6.70	8.26
Domestic chores	7.94	5.38	9.76	8.62
Driving	8.68	8.17	9.31	8.20
Leisure activities	7.68	6.26	8.36	8.85
Using new technologies	10.18	12.46	10.06	5.95
Social activities	7.99	11.44	6.30	5.27
Cinema, theater	2.96	3.65	2.56	2.59
Small-scale operations	5.15	2.35	5.76	9.19
Looking after others	2.75	2.46	2.85	3.11
Voluntary work	3.34	3.74	2.93	3.56
Artistic activities	3.89	5.76	2.61	3.28
Exhibitions, concerts, conferences	5.60	6.28	5.21	5.17
Holidays	3.54	2.16	3.94	5.36
Reading books	7.52	8.17	6.68	8.28
Pet care	2.94	3.87	2.63	1.79
Managing one’s bank account(s)	7.99	9.24	7.65	6.34

* Mean ± standard deviation. ** Percentages.

**Table 4:** Statistics for construct validity models of CRIq-Arabic.

	Model			
Variable	A	B	C	D
	**Fit statistics**			
*X* ^2^	0	45.37	41.48	39.52
*df*	0	23.00	21.00	17.00
*X*^2^/*df*		1.97	1.98	2.33
CFI	1	0.96	0.97	0.96
RMSEA		0.08	0.08	0.09
	**Loadings on cognitive reserve construct**			
CR-->education	0.42 **	0.46 **	0.44 *	0.35 *
CR-->working activity	0.66 *	0.63 **	0.64 *	0.59 *
CR-->leisure time	0.56 ^NA^	0.55 ^NA^	0.74 ^NA^	0.55 ^NA^
	**Correlations with other constructs**			
CR<->Gf		0.38 *	0.54 *	
CR<->Glr		0.24 *	0.39 *	
CR<->Gs		0.37 *	0.48 *	
	**Gf**	**Glr**	**Gs**	
	Loadings on other constructs			
**Model C**				
Education				
Working activity		−0.14		
Leisure time	−0.25			
**Model D**				
Education	0.27 *	−0.03	0.05	
Working activity	0.22	−0.08	0.12	
Leisure time	−0.02	0.03	0.24	

Gf: fluid intelligence. Glr: long-term memory encoding and retrieval. Gs: processing speed. CFI: Comparative Fit Index. RMSEA: root mean square error of approximation. CR: cognitive reserve. ***X*^2^**: chi-square. ***df***: degrees of freedom. ***X*^2^/*df***: critical ratio. * *p* < 0.05. ** *p* < 0.001. NA: not available

## Data Availability

The data presented in this study are available on request from the first and corresponding author.
